# Eye Care: I Care

**DOI:** 10.51866/mol.26-00027

**Published:** 2026-09-08

**Authors:** Hooi Chin Beh, Jazlan Jamaluddin

**Affiliations:** 1 Department of Primary Care Medicine, Faculty of Medicine, Universiti Malaya, Kuala Lumpur, Malaysia.

**Keywords:** Eye injuries, Corneal diseases, Vision disorders, Health promotion, Occupational health

The world had been dark for the past few days not only because my vision was blurred and painful but also because darkness entered my emotions when I faced losing something I took for granted.

Some experiences are as frightening as hearing that the cornea has been scraped away. When an injury affects the visual axis, fear becomes real. The uncertainty, helplessness and constant question ‘Will I see normally again?’ can be overwhelming.

Healthcare providers often remind patients to care for their eyes, advising safe screen habits, protective eyewear use and regular breaks. However, how often do they practise what they preach? Do they proactively discuss eye health during routine consultations or only when symptoms arise?

The truth is that most people do not fully appreciate vision until it is threatened. Vision makes it possible to witness a child’s smile, read, work and connect with the world. Because it serves faithfully, its value is often overlooked.

For weeks, recurrent corneal erosions following trauma left me with severe pain, redness, photophobia and reduced vision. Simple tasks became difficult, and the future felt uncertain. Then came the morning when sunlight returned. As dawn broke, rays of light entered the room. Outside, my children excitedly pointed to the sky as a rainbow appeared after the rain. For the first time in days, I could appreciate the colours again: the blue sky, the green trees and the vibrant arc across the horizon.

What once seemed ordinary suddenly felt extraordinary. That moment reminded me that vision is not merely a physical function. It is closely tied to independence, emotional well-being, productivity and quality of life. When vision is impaired, the impact extends far beyond the eye itself.

This reflection feels especially meaningful in June, during Cataract Awareness Month, a timely reminder to celebrate and appreciate the gift of vision while encouraging prevention, early detection and appropriate eye care.

Ironically, friends had long called me a workaholic. Their advice was simple: ‘You are straining your eyes too much’ or ‘You always wanted to have your work completed, not today or tomorrow, but yesterday’. However, modern medicine is increasingly practised through screens, such as electronic records, research and meetings. Much of it is meaningful, but when my vision faded, the irony became painfully clear. The screens that consumed my days became impossible to look at. Deadlines and performance targets suddenly lost importance when measured against sight.

This experience reminded me that productivity is a privilege supported by healthy vision. Regular eye examinations, sensible screen habits, adequate lubrication and prompt attention to symptoms are not trivial; they are investments in preserving one of the most precious senses.

Sometimes, it takes darkness to appreciate light.

Eye care: I care, and perhaps everyone should.

**Figure:**
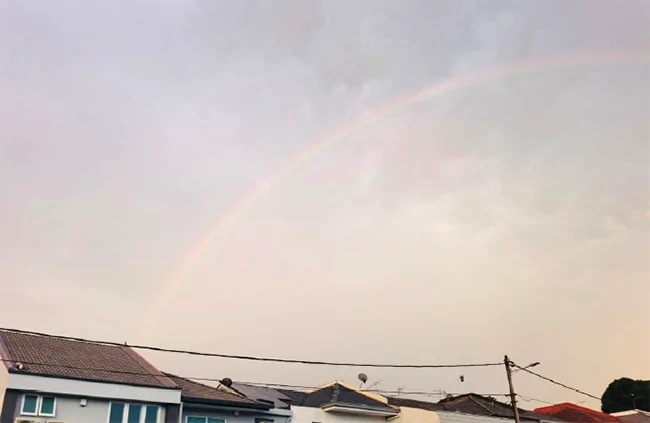
Photograph captured by the author on 5 June 2026, during the early stage of visual recovery, when she had regained the ability to perceivelight slowly.

